# Supplementary material for: Identification of photoperiod-induced specific miRNAs in the adrenal glands of Sunite sheep (Ovis aries)
Source: Front Vet Sci. 2022 Jul 22;9:888207. doi: 10.3389/fvets.2022.888207 (PMC9354845; doi:10.3389/fvets.2022.888207)
Supplement: Supplementary file 1 [file Data_Sheet_1.ZIP › Supplementary file/miRDeep2 file.zip!/miRDeep2 file/Know_Structure/SP42A2/oar-let-7f.pdf]

miRBase precursor : oar-let-7f
Total read count : 654991
oar-let-7f read count : 654843
remaining reads : 148

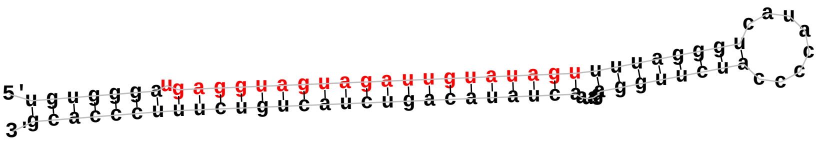

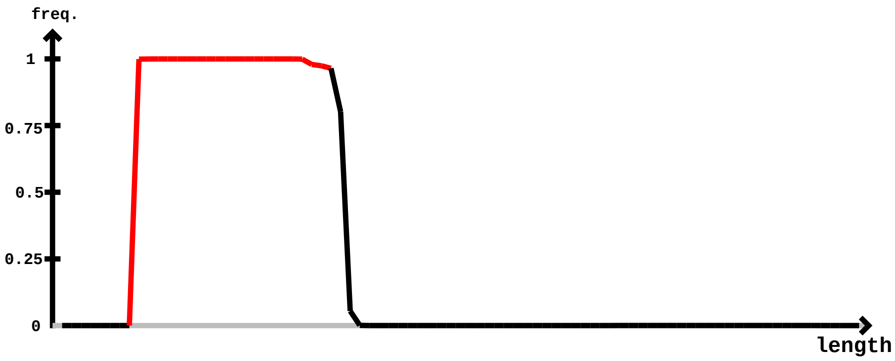

oar-let-7f

5'- ugugggaugagguaguagauuguauaguuuuagggucauaccccaucuuggagauaacuauacagucuacugucuuucccacg -3'	exp		
.((((((.((((((((((((((((((((((((((((........))))))))....)))))))))))))))))))))))))))))))).	reads	mm	sample
......Cugagguaguagauugu..	2	1	seq
......augagguaguagauuguau..	2	0	seq
......augagguaguagauuguauag..	1	0	seq
......augagguaguagauuguauagu...	4	0	seq
......augagguaguagauuguauaguu..	15	0	seq
......Gugagguaguagauuguauaguu..	6	1	seq
.......ugaAguaguagauugua...	4	1	seq
.......ugagguaguagauugua...	437	0	seq
.......ugagguagGagauugua...	3	1	seq
.......ugagguaguagauuguU...	5	1	seq
.......ugaggAaguagauugua...	1	1	seq
.......ugagguaguaAauugua...	2	1	seq
.......ugagguGguagauugua...	1	1	seq
.......ugagAuaguagauugua...	1	1	seq
.......ugagguaguagauuguau..	12948	0	seq
.......ugaAguaguagauuguau..	93	1	seq
.......ugagguaguUgauuguau..	18	1	seq
.......ugagguaguagauuguUu..	10	1	seq
.......ugagguaguagauugAaua...	1	1	seq
.......ugagguaguagauuguaua...	3260	0	seq
.......ugagguaguagauugAauag..	2	1	seq
.......ugagguaguagauuguauag..	6168	0	seq
.......ugagguaguagauugAauagu...	15	1	seq
.......ugagguaguagauuguauagu...	106433	0	seq
.......ugagguaguagauuguauaguu..	489279	0	seq
.......ugagguaguagauuguauaguuu...	33667	0	seq
.......ugagguaguagauuguauaguuG...	1579	1	seq
.......ugagguaguagauuguauaguuuu..	282	0	seq
.......ugagguaguagauuguauaguuGu..	119	1	seq
.......ugagguaguagauuguauaguuuua...	1	0	seq
........gagguaguagauuguau..	4	0	seq
........gagguaguagauuguaua...	1	0	seq
........gagguaguagauuguauag..	5	0	seq
........gagguaguagauuguauagu...	70	0	seq
........gagguaguagauuguauaguu..	296	0	seq

oar-let-7f

```
uguggga ugagguaguagauuguauagu uuuagggucauaccccaucuuggagauaacuauacagucuacugucuuucccacg
........gagguaguagauuguauaguuG.....................................................   1    1    seq
........gagguaguagauuguauaguuu.....................................................   22   0    seq
.........agguaguagauuguauagu.......................................................   1    0    seq
.........agguaguagauuguauaguu......................................................   7    0    seq
.........agguaguagauuguauaguuG.....................................................   10   1    seq
.........agguaguagauuguauaguuu.....................................................   2    0    seq
.........agguaguagauuguauaguuGu....................................................   55   1    seq
.........agguaguagauuguauaguuuu....................................................   1    0    seq
..........gguaguagauuguauaguu......................................................   7    0    seq
...........guaguagauuguauaguu......................................................   1    0    seq
............uaguagauuguauaguu......................................................   1    0    seq
............uaguagauuguauaguuuuagggu...............................................   1    0    seq
.............................uuagggucauaccccaucuugga...............................   1    0    seq
.............................uuagggucauaccccaucuuggaga.............................   4    0    seq
......................................................uaacuauacagucuacugucuu.......   1    0    seq
.........................................................cuauacagucuacugucuuu......   16   0    seq
.........................................................cuauacagucuacugucuuuc.....   27   0    seq
.........................................................cuauacagucuacugucuuucc....   98   0    seq
```
